# Breaking Up Sitting with Light-Intensity Physical Activity: Implications for Shift-Workers

**DOI:** 10.3390/ijerph14101233

**Published:** 2017-10-16

**Authors:** Grace E. Vincent, Sarah M. Jay, Corneel Vandelanotte, Sally A. Ferguson

**Affiliations:** School for Health, Medical and Applied Sciences, Central Queensland University, Wayville 5034, Australia; s.jay@cqu.edu.au (S.M.J.); c.vandelanotte@cqu.edu.au (C.V.); sally.ferguson@cqu.edu.au (S.A.F.)

**Keywords:** night-shift, non-communicable disease, sedentary behaviour, sitting breaks, shift-work

## Abstract

Prolonged sitting, restricted sleep, and circadian disruption are all independent risk factors for non-communicable diseases. Previous research has demonstrated that breaking up sitting with light-intensity physical activity has clear benefits for the health of day workers, but these findings may not apply in the presence of sleep restriction and/or circadian disruption—both of which are commonly experienced by shift-workers. Specifically, sleep restriction, and circadian disruption result in acute physiological changes that may offset the benefits of breaking up sitting. This commentary will explore the potential benefits of breaking up sitting for health, work performance, and subsequent sleep in shift-workers. Future areas of research designed to understand the mechanisms by which prolonged sitting and shift work impact worker health and safety and to support the design of effective occupational health and safety interventions are proposed.

## 1. Introduction

Physical inactivity is the leading risk factor for a number of non-communicable diseases (e.g., cancer, type 2 diabetes, and heart disease) [1] with an annual global economic burden (health care, lost productivity) of approximately $67 billion [2]. It is estimated that up to 60% of adults are insufficiently active for health [3]. To prevent non-communicable disease, most research has traditionally focused on the potent and widespread benefits of engaging in moderate-to-vigorous physical activity (MVPA). However, focusing exclusively on promoting MVPA, which at most accounts for ~5% of time during the day, limits the potential to optimise health benefits of other daily activities, such as those involving light-intensity physical activity (e.g., standing, slow walking), sitting, and sleep, that make up ~95% of the day [4]. 

In the last decade, a substantial amount of evidence has demonstrated that time spent sitting is associated with increased non-communicable disease risk [5,6] and all-cause mortality [7,8]. Importantly, the adverse health outcomes associated with prolonged sitting are independent of habitual physical activity levels [9]. Therefore, those who are regularly engaged in MVPA but also sit for prolonged periods may still be at increased risk of non-communicable disease [10]. To reduce the adverse effects of prolonged sitting, research over the last five years has focused on breaking up prolonged sitting at regular intervals (e.g., every 20–30 min) with short bursts (e.g., 2–3 min) of light-intensity physical activity [11]. Studies have observed improvements in metabolic health [12], self-reported fatigue [13], and reductions in all-cause mortality [14]. In addition, the simple transition from a seated to a standing posture every 30 min across a work day reduces fatigue, lower back discomfort, and maintains work productivity, compared to seated-only work [15]. The majority of this research has been conducted in physically inactive, type 2 diabetic, or young habitually physically active participants [11]. However, few studies have investigated the impact of breaking up sitting in shift working populations [16], a population that is already at increased risk of chronic ill-health [17,18,19]. 

Shift work is defined as “work arrangements outside conventional daytime hours, and can include fixed early morning, evening, and night work, as well as roster work and rotating three shift work” [17]. Nearly 20% of the Western workforce is currently involved in rotating shifts or night work, with the prevalence of shift work arrangements predicted to increase [20]. Shift work is adversely associated with numerous non-communicable diseases, including type 2 diabetes, cardiovascular disease, coronary heart disease, stroke, and cancer [17,18,19]. While there is generally a poor understanding of the mechanisms by which shift work leads to non-communicable disease [17], sleep restriction and circadian disruption (commonly associated with shift work [21]) have both been implicated [17]. Contributing also to shift workers’ risk for ill-health may be time spent in occupational sedentary behaviours. Sedentary behaviour is defined as any waking behaviour characterised by an energy expenditure ≤1.5 metabolic equivalents (METs), while in a sitting, reclining, or lying posture [22], (i.e., watching TV, using a computer, and sitting in cars). Compared to day workers, shift workers spend comparable amounts of time engaged in both physical activity and sedentary behaviours during leisure-time [23] but may spend up to 39.8% of time engaged in occupational sedentary behaviour compared to 30.8% in day workers [23]. Further, compared to day workers, night shift workers spend 4.3% more time at work in uninterrupted sedentary periods of >30 min [23].

Given the complexities associated with shift worker ill-health compared to day workers and the increased exposure to occupational sedentary behaviours, this commentary will explore the appropriateness and relevance of breaking up sitting with light-intensity physical activity in shift work environments. The effectiveness of breaking up sitting with physical activity in sleep-restricted and/or circadian-disturbed individuals will be discussed. In addition, there are known consequences of sleep restriction and circadian disruption for cognitive function [24,25] and accident and error risk, [26,27,28]. These data, combined with evidence that physical activity can improve short-term cognitive performance [13,29] and positively influence sleep [30], warrants a critical appraisal of whether breaking up sitting with physical activity may benefit work performance and sleep, which will follow. Finally, future research directions for investigating breaking up sitting in shift work environments will be explored. 

## 2. Are the Benefits of Breaking Up Sitting with Physical Activity as Effective in Sleep-Restricted and/or Circadian-Disturbed Individuals?

Sleep restriction, circadian disruption, and prolonged sitting are all characteristic of shift work arrangements and detrimental for shift workers’ health and safety [17,23,31]. Importantly, these factors often occur in combination [23,32]. However, the problem for managing health and safety risks in shift work settings is that mitigation strategies have largely been tested in isolation, rarely taking into account the potential interaction effects of sleep restriction, circadian disruption, and prolonged periods of sitting. For example, studies investigating breaking up sitting with physical activity [11,33] have not tested individuals’ experiencing sleep and/or circadian disruption. This leaves a gap in our understanding of the health benefits of breaking up sitting for shift workers experiencing circadian disruption or sleep restriction. 

Sleep restriction and circadian disruption in shift workers can be a consequence of work hours (e.g., sleeping and waking at biologically inappropriate times, extended hours, overtime) or non-work factors (e.g., illness, caring duties) [34]. The physiological desynchrony caused by circadian disruption may mean that breaking up sitting with short bouts of physical activity does not have the same effect in shift workers experiencing circadian disruption, as has been reported in those working during the day. Further, while breaking up sitting with physical activity has been shown to be beneficial for reducing the risk factors of non-communicable diseases [35], studies have not controlled or reported prior sleep duration [11,33]. This is important, as short sleep duration is associated with negative changes to the same metabolic and inflammatory markers that are positively influenced by breaking up sitting. As such, it is possible that short sleep durations may attenuate the positive impacts of breaking up prolonged sitting [36]. Therefore, more physical activity during breaks may be required (either longer durations of physical activity and/or higher intensity, e.g., MVPA rather than light-intensity physical activity) to get the same effect on physiological parameters in shift workers. 

## 3. Can Breaking Up Sitting with Physical Activity Improve Shift Workers’ Performance and Sleep?

Sleep restriction and circadian disruption increases sleepiness, reduces alertness, and is a risk factor for impaired cognitive performance, and increased likelihood of accidents [12,28,29,30]. Sleep restriction impairs many aspects of work performance, including motor skills, concentration, memory, and learning [37]. In addition, declines in cognitive performance can lead to higher rates of fatigue-related errors and accidents. The risk of occupational accidents is up to 60% higher for non-day shift workers [26,27]. Road and workplace accidents related to excessive sleepiness, to which shift work is a significant contributor, are estimated to cost up to $93 billion per annum in the United States, and fatigue is believed to contribute to 20% of road accidents in Australia [27]. Sleep restriction also leads to adverse behavioural outcomes such as irritability, negative mood, reduced communication, and ability to cope with work demands [38]. Further, decision-making ability declines and risk-taking behaviours increase [39,40]. It is also of concern that sleep-restricted individuals may not be aware of their own cognitive performance declines [24]. Management of health and safety risks associated with the consequences of shift work remains a significant challenge for industry, regulators, and individuals. 

Task rotation and in-shift breaks are established risk management controls, and are prescribed as part of fatigue risk management policies, to mitigate against cognitive performance impairment and reduced alertness in shift workers [41]. While the benefits for alertness and work performance of in-shift breaks are well known, research has focused on the length of break or inclusion of a nap [18], but the underlying mechanisms that explain these benefits are not well understood. For example, very little is known about what aspect of a rest break contributes to improving work performance and alertness (e.g., cognitive rest, sleep, and physical activity). Beneficial effects of exercise on cognitive performance have been observed following a single bout of exercise [29] and a recent pilot study found breaking up sitting may be an effective acute fatigue countermeasure [13], suggesting that at least light-intensity physical activity may mediate pathways involved in mental fatigue and cognition. Further research is needed to determine whether breaking up sitting with physical activity benefits performance in the workplace.

Epidemiological studies have shown that exercise is consistently associated with better sleep, across many demographics [42,43]. Further, experimental studies highlight that acute and regular exercise is associated with improvements in sleep [30]. Possible mechanisms by which exercise improves sleep include increases in growth hormone and mood, as well as decreases in insulin resistance, vagal activity and inflammation [30]. Further, the rapid decline in core body temperature following exercise increases the likelihood of sleep onset and may facilitate entry into deeper sleep stages [44]. In addition, day time exercise is frequently included into standard sleep hygiene practices [45]. However, the impacts of prolonged sitting and breaking up sitting on sleep outcomes are unclear. A recent study observed improvements in subjective but not objective sleep quality measures in hypertensive adults that used sit–stand desks compared to those who were continuously seated [46]. Therefore, further studies are needed to explore whether breaking up sitting with physical activity has beneficial effects for subsequent sleep.

## 4. Benefits of Future Research Investigating Breaking Up Sitting in Shift Workers

Prolonged sitting, sleep restriction, and circadian disruption are workplace health and safety hazards. Mitigating the risks of these hazards requires an understanding of the underlying mechanisms impacting health and safety outcomes, in order to design and test appropriate control measures. In isolation, these hazards are quite well understood [11,12], but to date they have not been studied in combination in controlled settings. The proliferation of standing desks, treadmill work stations and walking meetings in many workplaces is a testament to a growing emphasis on increasing physical activity to manage the work health and safety risks associated with sitting. However, mechanisms by which breaking up sitting with physical activity mitigates risk in day workers may not be the same in workers who experience sleep restriction and circadian disruption such as shift workers. Therefore, it is timely to investigate whether these factors impact the health strategies associated with breaking up sitting.

Research is needed to determine how aspects of shift work interact with breaking up sitting, and whether breaking up sitting strategies could also be beneficial to shift workers in terms of work performance and safety. To test this, experimental studies are needed to contribute foundational knowledge about the underlying mechanisms by which breaking up sitting impacts physiological markers and work performance in sleep and circadian-disrupted individuals. One example is testing the optimal length of break and intensity of physical activity required to benefit health, in shift working and non-shift working populations. Future studies could also determine the health and performance impacts of different combinations of activity (e.g., sedentary versus active) and sleep (e.g., 5-h versus 9-h sleep opportunities). 

New knowledge generated from experimental studies will contribute to the evidence base on which tailored and effective workplace interventions can be designed and tested. Translation to systems of work that better support the health of shift workers who sit and do not get sufficient sleep are needed [47]. For example, infrastructure that increases physical activity (e.g., treadmill desks) could be implemented at work, combined with improved sleep hygiene in the home (e.g., blackout curtains for night shift workers sleeping during the day). Previous research has demonstrated that workplace interventions have typically focused on reducing sitting time and increasing work standing time [48]. While small improvements in biomarkers of cardiometabolic risk following such workplace interventions have been observed [49], the potential benefits for workers at high risk for cardiovascular disease (e.g., shift workers) may be greater. Flexible work environments that support sit–stand–physical activity transitions should be provided, given that excessive standing is also associated with increased cardiometabolic risk [50]. The potential impacts of effective interventions on workplaces and the broader economy are wide-reaching and may include improved productivity and reduced rates of absenteeism or presenteeism. However, further research is needed to examine to what extent these benefits ensue and whether workplaces are supportive of creating environments that allow reductions in sitting time. Existing evidence indicates that low-cost approaches to reduce workplace sitting are perceived to be feasible, but other factors such as work demands and organisational social contexts may act as barriers [51]. Therefore, it is recommended that building a supportive organisational culture and raising awareness of the adverse health effects of prolonged sitting are essential for improving individual level and organisation-level behaviour change [51]. 

Measurement of sleep, sitting breaks, and physical activity should be standardised to ensure a consistent approach to research and comparability between studies [47]. In laboratory settings, polysomnography, the gold standard measure of sleep, integrates measures of brain activity, eye movement, muscle activity, and cardiac activity, to identify sleep and wake, as well as, individual sleep stages [52]. Activity monitoring (actigraphy, accelerometry) provides an objective, non-invasive practical free-living alternative to polysomnography [53]. Activity monitors indirectly assess sleep by sensing motor activity at the wrist and use valid algorithms to distinguish sleep from wakefulness [54,55]. These devices are useful for collecting data continuously for long periods of time and can permit concurrent measurement of sedentary behaviour and physical activity [56]. However, to measure sit–stand transitions or brief sitting breaks, devices that include an inclinometer and worn on the thigh may be more valid [57]. Clear reporting of activity monitor data (e.g., type, intensity, location) is crucial in the evaluation and replication of experimental and intervention studies [58]. In workplaces, commercially available sleep and activity trackers (e.g., Garmin) could be provided to monitor worker behaviour in real time.

## 5. Conclusions

Time spent sitting, sleep restriction, and circadian disruption are all independent risk factors for non-communicable diseases. However, we understand very little about how these risk factors interact with one another. Furthermore, sleep restriction and circadian disruption also impact on alertness and work-related performance and safety, with significant associated costs. It is possible that in-shift breaks that involve breaking up sitting with physical activity may have considerable benefits for health, work performance, and sleep of shift workers. Therefore, understanding the combined impact of these behaviours on non-communicable disease risk, as well as how breaking up sitting with physical activity may influence other aspects of worker health and safety (e.g., cognitive performance, sleep) is critical. In order to design appropriate risk mitigation strategies for prolonged sitting in shift workers, both foundational experimental studies and interventions aimed at designing work patterns and practice are needed to alleviate the health and safety burden on shift workers worldwide.
